# Supplementary material for: Content-rich biological network constructed by mining PubMed abstracts
Source: BMC Bioinformatics. 2004 Oct 8;5:147. doi: 10.1186/1471-2105-5-147 (PMC528731; doi:10.1186/1471-2105-5-147)
Supplement: Additional File 2 — The original results of the above study (non-essential files are deleted to keep the file size under the limit set by BMC bioinformatics). [file 1471-2105-5-147-S2.bz2 › chilibotAdditionalFile2/dip50/638ID8114095E11025/html/TRXA_TRXB.html]

 


 **TRXA** and **TRXB** 
  
Found 679 abstracts in PubMed, retrieved 50.  
 

 What does Google say? 
 PDF only 
| .edu only 

---

**Interactive relationship** (e.g. stimulation, inhibition, etc)

**Inhibitory relationship**- Peroxiredoxin 3 Prdx3 is a mitochondrial member of the antioxidant family of thioredoxin  [ **TRXA** ]  peroxidases that uses mitochondrial thioredoxin  [ **TRXA** ]  2 Trx2  [ **TRXB** ]  as a source of reducing equivalents to scavenge hydrogen peroxide H 2 O 2.  Ref: 12861054 Mol Cancer Res, 2003
**Stimulatory relationship**- Thioredoxin  [ **TRXA** ]  Trx  [ **TRXA** ]  expression is increased in several human primary cancers and the Trx  [ **TRXA** ]  Trx  [ **TRXA** ]  reductase TrxR  [ **TRXB** ]  system therefore provides an attractive target for cancer drug development.  Ref: 12569302 Anticancer Drugs, 2003
**Neutral relationship**- Trx  [ **TRXA** ]  expression also correlated with gammaGCSh P< ; 0.001 and gammaGCSl P=0.012 and TrxR  [ **TRXB** ]  with gammaGCSh P< ; 0.001 but not with gammaGCSl immunoreactivity P=0.744.  Ref: 12499089 Lung Cancer, 2003

**Non-interactive relationship** (e.g. studied together, co-existance, homology, etc.)

- Necrotic tumors were more often immunopositive for Trx  [ **TRXA** ]  p = 0.001 and TrxR  [ **TRXB** ]  p = 0.02 and AOE expression was generally higher in mitotically active tumors.  Ref: 12744469 Brain Pathol, 2003
- Western blot showed that temocapril enhanced cytosolic redox regulatory protein TRX  [ **TRXA** ]  expression, but neither mitochondrial TRX2  [ **TRXB** ]  nor antioxidant enzymes, such as copper zinc superoxide dismutase Cu Zn SOD or manganese superoxide dismutase Mn SOD expression, was increased by the preconditioning treatment.  Ref: 12870672 Mol Cell Biochem, 2003
- Within diffuse astrocytomas only Trx  [ **TRXA** ]  p = 0.0001 and TrxR  [ **TRXB** ]  p= 0.04 significantly associated with increased malignancy grade.  Ref: 12744469 Brain Pathol, 2003
- We studied the expression of antioxidant enzymes AOEs and related proteins manganese superoxide dismutase MnSOD, thioredoxin  [ **TRXA** ]  Trx  [ **TRXA** ] , thioredoxin  [ **TRXA** ]  reductase TrxR  [ **TRXB** ] , and the catalytic GLCL c and regulatory GLCL r subunits of glutamate cysteine ligase gamma glutamylcysteinesynthetase in 433 astrocytomas.  Ref: 12744469 Brain Pathol, 2003
- However, inhibition of thioredoxin  [ **TRXA** ]  reductase TrxR  [ **TRXB** ]  by a low dose of gold in the form of aurothioglucose led to induction of hepatic HO activity.  Ref: 12706824 FEBS Lett, 2003
- Normal bronchial epithelium was positive for MnSOD, heavy and light subunits of gammaGCS, CAT and Trx  [ **TRXA** ]  and TrxR  [ **TRXB** ] .  Ref: 12499089 Lung Cancer, 2003
- Diffuse astrocytomas showed more intense staining for Trx  [ **TRXA** ]  p = 0.002, TrxR  [ **TRXB** ]  p = 0.004, GLCL c p = 0.001, GLCL r p = 0.04 and MnSOD p = 0.01 than pilocytic astrocytomas.  Ref: 12744469 Brain Pathol, 2003
- Expression of MnSOD was found in 91%, Trx  [ **TRXA** ]  in 46%, TrxR  [ **TRXB** ]  in 66%, GLCL c 73% and GLCL r in 89% of the cases.  Ref: 12744469 Brain Pathol, 2003
- The lack of thioredoxin  [ **TRXA** ]  reductase  **trxB**  and glutathione reductase gor in Origami B DE3 allowed formation of disulfide bridges in the cytosol.  Ref: 12694197 Eur J Biochem, 2003
- The primary structure of the alpha subunit demonstrates a high similarity to the structures of thioredoxin  [ **TRXA** ]  reductase TrxR  [ **TRXB** ]  from many prokaryotes, especially Pseudomonas aeruginosa identity 85%, positive 91%, Vibrio cholerae identity 73%, positive 85%, and Escherichia coli identity 71%, positive 83%.  Ref: 12686150 Biochim Biophys Acta, 2003
- Cellular defense systems against reactive oxygen species ROS include thioredoxin  [ **TRXA** ]  reductase TrxR  [ **TRXB** ]  and glutathione reductase GR.  Ref: 12715897 Biol Chem, 2003
- RESULTS Temocapril enhanced cytosolic redox regulatory protein TRX  [ **TRXA** ]  expression, but neither mitochondrial TRX2  [ **TRXB** ]  nor antioxidant enzymes, such as copper zinc superoxide dismutase Cu Zn SOD or manganese superoxide dismutase Mn SOD expression, was up regulated by the preconditioning treatment.  Ref: 12882710 Zhonghua Nei Ke Za Zhi, 2003
- There was a significant correlation between expression of gammaGCSh and gammaGCSl P=0.034 and Trx  [ **TRXA** ]  and TrxR  [ **TRXB** ]  P=0.037.  Ref: 12499089 Lung Cancer, 2003
- In this study, mutants in the trxA1 and trxA2 genes encoding Trx1  [ **TRXA** ]  and Trx2  [ **TRXB** ] , respectively, and in the tpx and bcp genes, which encode the remaining two members of the H. pylori peroxiredoxin family, were constructed in order to determine their roles in resistance to damage by reactive oxygen and nitrogen species.  Ref: 12576586 Microbiology, 2003
- Helicobacter pylori possesses two distinct thioredoxin  [ **TRXA** ]  proteins Trx1  [ **TRXA** ]  and Trx2  [ **TRXB** ]  which may play important roles in the ability of this bacterium to survive oxidative stress.  Ref: 12576586 Microbiology, 2003
- Data from the present data further showed that while Trx  [ **TRXA** ]  and Trx  [ **TRXA** ]  reductase TrxR  [ **TRXB** ]  in cytoplasm decrease with age in AL fed rats, CR prevents these decreases.  Ref: 12742531 Exp Gerontol, 2003
